# Cost of implementing a community-based primary health care strengthening program: The case of the Ghana Essential Health Interventions Program in northern Ghana

**DOI:** 10.1371/journal.pone.0211956

**Published:** 2019-02-07

**Authors:** Edmund Wedam Kanmiki, James Akazili, Ayaga A. Bawah, James F. Phillips, John Koku Awoonor-Williams, Patrick O. Asuming, Abraham R. Oduro, Moses Aikins

**Affiliations:** 1 Regional Institute for Population Studies, University of Ghana, Legon, Accra, Ghana; 2 Navrongo Health Research Centre, Ghana Health Service, Upper East Region, Ghana; 3 Department of Population and Family Health, Mailman School of Public Health, Columbia University, New York, New York, United States of America; 4 Policy, Planning, Monitoring, and Evaluation Division, Ghana Health Service Head Quarters, Accra, Ghana; 5 University of Ghana Business School, Legon, Accra, Ghana; 6 School of Public Health, College of Health Sciences, University of Ghana, Legon, Accra, Ghana; University of the Western Cape, SOUTH AFRICA

## Abstract

**Background:**

The absence of implementation cost data constrains deliberations on consigning resources to community-based health programs. This paper analyses the cost of implementing strategies for accelerating the expansion of a community-based primary health care program in northern Ghana. Known as the Ghana Essential Health Intervention Program (GEHIP), the project was an embedded implementation science program implemented to provide practical guidance for accelerating the expansion of community-based primary health care and introducing improvements in the range of services community workers can provide.

**Methods:**

Cost data were systematically collected from intervention and non-intervention districts throughout the implementation period (2012–2014) from a provider perspective. The step-down allocation approach to costing was used while WHO health system blocks were adopted as cost centers. We computed cost without annualizing capital cost to represent financial cost and cost with annualizing capital cost to represent economic cost.

**Results:**

The per capita financial cost and economic cost of implementing GEHIP over a three-year period was $1.79, and $1.07 respectively. GEHIP comprised only 3.1% of total primary health care cost. Health service delivery comprised the largest component of cost (37.6%), human resources was 28.6%, medicines was 13.6%, leadership/governance was 12.8%, while health information comprised 7.5% of the economic cost of implementing GEHIP.

**Conclusion:**

The per capita cost of implementing the GEHIP program was low. GEHIP project investments had a catalytic effect that improved community-based health planning and services (CHPS) coverage and enhanced the efficient use of routine health system resources rather than expanding overall primary health care costs.

## Introduction

Four decades ago, the Alma-Ata declaration on primary health care enjoined countries to make health care accessible, affordable and culturally situated [1–4]. Low and middle-income countries responded to Alma Ata by adopting community-based strategies designed to improve both geographic and financial access to basic health care in culturally acceptable ways [5–8].

In Ghana, this has taken the form of a national program known as the Community-based Health Planning and Services (CHPS) initiative [9]. CHPS is a community-based approach to delivering basic preventive and curative health care services to rural communities by stationing nurses called Community Health Officers (CHOs) in defined village community locations [10, 11]. Rather than relying solely on the provision of healthcare in facilities, CHPS workers engage in community outreach and doorstep care[12].

CHPS originated in the early 1990’s in response to debates on the practicality of achieving the Alma Ata goal of health for all by the year 2000. To resolve such debates, Ghana’s Ministry of Health constituted health research stations in each ecological zone of Ghana. Accordingly, the Navrongo Health Research Center (NHRC) was established in 1992 as a government-based research site operating under the Ghana Health Service. Located in Sahelian northern Ghana, the NHRC was provided with a mandate to develop evidence-based primary health service strategies. The locality where its research was conducted represented an ideal setting for policy relevant research. Its study districts are located in one of the most impoverished and remote regions of Ghana, where pervasive poverty intersects with high illiteracy and social customs associated with marriage, kinship and family building that were governed more by indigenous traditions than by awareness of modern health care[13]. This setting, therefore, represented an unpromising locality for which any successful experimental research could not be dismissed on the pretext of being a by-product of favorable circumstances and economic trends [14, 15]. The presence of a Health and Demographic Surveillance System (HDSS) for monitoring the mortality, morbidity, fertility and other dynamics in the area has greatly enhanced the contribution of Navrongo research to policy deliberations [16].

The initial Navrongo pilot study, spanning 1994–1996, was a three village micro-pilot which combined implementation with social research for gauging the reactions of the community to primary health care operations [13]. This application of “participatory planning” generated strategies for community engagement, volunteer recruitment and deployment, and nursing services [17]. Since the relative merits of community nurse posting and volunteer deployment was not resolved by a pilot, operations were scaled up to a 36 community district-wide plausibility trial that tested the impact of community-based nursing services, volunteer-focused care, and combining nurse and volunteers relative to a comparison condition that involved existing sub-district health services alone[18–20].

Results of the “Navrongo Experiment” showed that when trained professional community nurses were assigned as community resident primary health care providers, child mortality was reduced by 50% and maternal mortality by 40% within five years. In communities where volunteers were made responsiblee for male engagement and community mobilization, total fertility rate (TFR) reduced by nearly a birth within five years of implementation [12, 14, 18, 21, 22].

The Navrongo approach to service delivery was replicated in the Nkawanta District of the Volta region of Ghana from 1999–2002. The replication also yielded success in a number of health indicators including family planning utilization which increased in three years from less than 4% to 14%. Odds of receiving antenatal care improved by five-fold while postnatal care odds were also four times greater for women in communities exposed to the program [23]. The Nkwanta replication trial provided evidence that the Navrongo approach was not only effective, but it could also be scaled-up across the country.

With the goal of scaling up successful Navrongo interventions, the Ghana Health Service established the Community-based Health Planning and Services (CHPS) as a national policy in 1999 implemented from 2000. CHPS monitoring evidence compiled over the 2000 to 2008 period revealed that by mobilizing rural villages to develop systems for providing primary health care, the Navrongo approach could save lives, reduce fertility and accelerate the achievement of the then Millennium Development Goals while paving the way for achieving the current health-related Sustainable Development Goals [24, 25].

However, monitoring showed that this impact was limited to communities where CHPS operations were functional [26]. The implementation of CHPS services has been impeded by a variety of service delivery, manpower, communication, logistics, resource management, and leadership bottlenecks [26]. Critical among the barriers has been the lack of health sector budgetary commitments to start-up CHPS. Financing for CHPS start-up is funded only sparingly by district assemblies and the global community[21]. The World Bank, the European Union, and some bilateral donors commit resources to flexible decentralized common fund revenue pool of district assemblies [21]. While these pools of resources could be directed for CHPS facility construction, district health managers must demonstrate the overarching need for such investment amidst competing demands on the development budget from other sectors. In the interim, through optimal community mobilization, donated material and volunteer labor can be harnessed for constructing temporary CHPS posts. However, implementation studies showed that most district health managers lacked the capacity to galvanize local political support and community engagement for CHPS scale-up [21, 26].

In the view of these managers, CHPS functionality depended upon the construction of health posts where services could be provided and nurses could reside. Designs for these facilities were not standardized, but costs typically exceeded $20,000 for material and labor alone. In the typical district, implementation was delayed until resources for these initial costs could be found. Moreover, several proven interventions were not yet introduced into the CHPS program [21].

In response to this monitoring evidence, Ghana’s Ministry of Health launched a review of the CHPS program in 2009 which aimed to clarify the operational and policy barriers to effective CHPS scale-up[27]. Results of this review provided a set of systems development needs and an agenda that brought forth the operational design of a project known as the Ghana Essential Health Interventions Program (GEHIP) [21]. The implementation of GEHIP was effective in scaling-up CHPS coverage from an initial 20% to 100% coverage of the rural population in intervention districts compared to non-intervention districts which had recorded a rise from 35% to 50% coverage of the rural population [17]. Also, results of the impact of GEHIP on child mortality using difference-in-difference estimates for the incremental effect of GEHIP contrasting baseline and end-line child mortality records for both intervention and comparisons areas shows that GEHIP package of interventions reduced infant mortality by almost half (DiD HR = 0.52, 95% CI = 0.28,0.98; p = 0.045) [28]. The success of GEHIP, therefore, merits appropriate documentation of the cost associated with implementing strategies for achieving such success. The goal is to address the need for information that would be essential to any initiative in the future that may seek to replicate GEHIP or to scale it up nationwide [29].

### Background of GEHIP intervention

GEHIP was a health system strengthening and research program designed as a plausibility trial for testing the hypothesis that a set of interventions could improve district leadership, marshal the scale-up and impact of CHPS, strengthen primary health care by accelerating CHPS implementation and improve the utilization of primary health care to improve health and under-five survival.

GEHIP was implemented to provide practical guidance for accelerating the expansion of community-based primary health care and introducing improvements in the range of services community workers can provide [17]. The GEHIP strategy included the introduction of a series of training and technical assistance programs aimed at strengthening the capacity of the health system. The GEHIP programs were coordinated by experienced regional CHPS coordinators with occasional support of other public health specialist in pediatrics, health systems, and leadership. There was no shortage of nursing staff for scaling-up CHPS operations, however, lack of health facilities to post trained nurses in most communities/villages was a challenge [21]. In addition, there was limited understanding of strategies for addressing revenue needs for health post construction [21]. Available evidence shows that where CHPS implementation was rapid, health managers developed community engagement approaches that led to low-cost volunteer construction of community health posts [21]. Model implementation of this kind presented an avenue for demonstrating the popularity of CHPS and generating grassroots political support that could foster district assembly commitment to financing CHPS post construction[30].

GEHIP compiled these observations into a coherent strategic framework for community-based primary healthcare strengthening that emphasized the importance of enhancing district leadership capacity for effective systems functioning. GEHIP posits that developing leadership, information for decision-making, budgeting, logistics, training, and worker deployment would enhance the provision of health services at community locations and impact on the survival of children. Maternal and child health interventions were also added, and increased support was provided to system structures to enhance overall effectiveness. Sets of health systems strengthening activities were pursued which involved community-engagement for organizing the provision of the WHO recommended regimen for integrated management of childhood illness [31]. Frontline workers were trained and adequately equipped to deal with the lead causes of neonatal morbidity and mortality. A comprehensive referral service was developed for GEHIP districts that involved the promotion of facility-based delivery, the organization of a communication system, and a process of convening community engagement for sustaining social support for referral operations [21, 32]. While the GEHIP interventions were being implemented, a region wide (in both intervention and non-intervention districts) program of health worker training in the WHO recommended care of the sick newborn [33] was also implemented. However, GEHIP interventions were aimed at improving program access to WHO recommended modalities and procedures by implementing a trial package of leadership, community engagement, and emergency health service interventions.

GEHIP was implemented in the Upper East Region (UER) of northern Ghana by the Upper East Regional Health Directorate of the Ghana Health Service (GHS) with technical assistance from the Columbia University Mailman School of Public Health (MSPH) and University of Ghana School of Public Health (UGSPH). Navrongo Health Research Center (NHRC) was responsible for the monitoring and evaluation of the GEHIP project.

**Fig 1:**
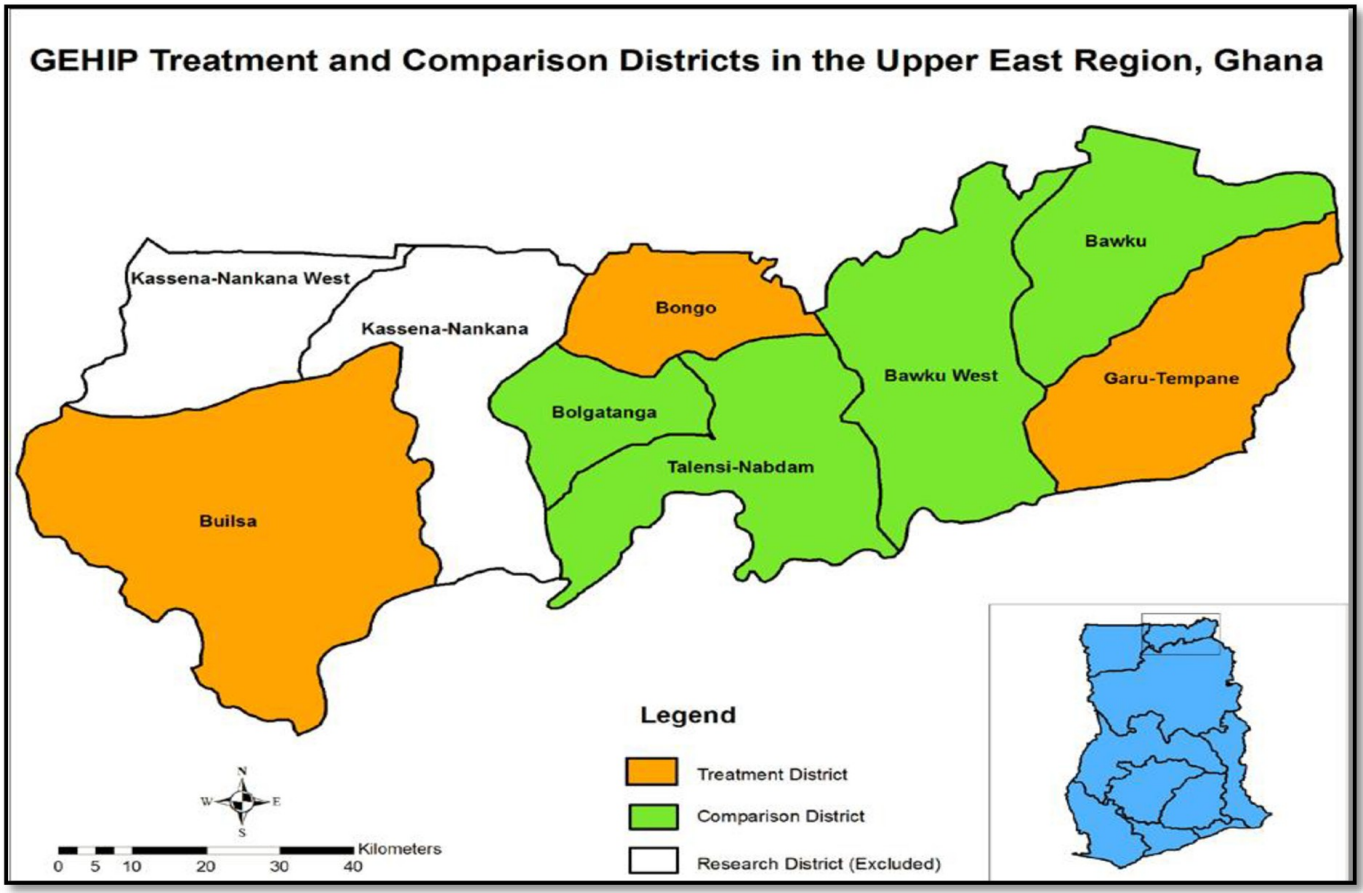
Map of Upper East Region showing GEHIP intervention and comparison districts.

The original design of GEHIP specified three purposefully selected intervention districts (Builsa, Bongo, and Garu-Tempane) and four comparison districts (Bolgatanga, Bawku East, Bawku West, and Talensi-Nabdam). Both treatment and comparison districts were chosen because of their geographic isolation and socioeconomic deprivation. At the inception of GEHIP, these districts were ranked among the poorest 5% of Ghana’s districts with a per capita income level of about a quarter of the level of Ghana as a whole, with their local economies dominated by rain feed substance agriculture, high illiteracy and pervasive poverty were key characteristics of these districts [21, 34]. As a plausibility trial, the intervention districts were purposively selected and were observed to be more geographically isolated and health deprived in terms of infrastructure and staff compared to the non-intervention districts. This was done to ensure that any success that might emanate from the interventions could not be dismissed on the basis of favorable conditions. Owing to the potentially confounding effects of successful NHRC trials of health service interventions, two UER districts (Kassena-Nankana East and West) which were the initial pilot site of the “Navrongo Experiment” were excluded from GEHIP (Fig 1).

More details of the GEHIP project interventions can be found in previous publications [17, 21, 28, 35–37]. Project monitoring evidence has shown that GEHIP was effective in accelerating CHPS scale-up. GEHIP achieved 100% CHPS coverage in intervention districts [17], mainly by demonstrating ways to motivate community engaged construction of health posts as well as community support for a program of emergency care [17, 28]. Developing district leadership for managerial actions that build community engagement strategies was central to GEHIP’s operational design [21]. If community engagement was fully functioning, and the demand for CHPS implementation was well promoted, volunteer labor, donated construction material, and traditional leadership support could be marshaled for the task of constructing interim facilities for CHPS service activities. This strategy of interim facility development could permit CHPS services to begin without delaying operations until fully financed construction could be completed. This paper provides an analysis of the cost of implementing GEHIP with the goal of facilitating the implementation of future policy initiatives that invest resources in the scale-up of community-based primary health care services.

## Methods and materials

### Study design

The widely used step-down allocation approach described by Shepard et al [38] and used by other researchers for costing health care services [39–42] was used in this study. We adopted five WHO health system building blocks as cost centers. These are i) the development of health human resources, ii) health service delivery, iii) the provision of medicines & vaccines, iv) health information, and v) leadership/governance building blocks. We described these cost centers by specifying input procedures and corresponding costs implied by each respective building block. All intermediate and final costs were calculated according to the total and unit cost of implementing GEHIP and sustaining routine primary healthcare costs. Table 1 provides a description of the cost categories allocated to each of the cost centers.

**Table 1:** Costs items considered, classified into WHO health system building blocks.

Cost categories (cost centers)	Cost items considered under each category
Human Resources	Salaries of staff, other benefits, allowances (over time, clothing etc.), other incentives, seminars, workshops and conferences, school fees for staff on further studies, per diems, travel allowance, flights, accommodation).
Health Service Delivery	Cost of stationery, fuel, routine maintenance of vehicles and general equipment, cleaning materials and utilities (e.g., electricity, water and gas), furniture and equipment for service delivery.
Medicines & Vaccines	Medicines, non-medical consumables, other medical and laboratory supplies (e.g., thermometers, BP cuffs, gloves, mask, test kits, slides etc).
Health Information	Phone credit and internet models, software (e.g. antivirus, word etc.), printing and copying. Other communication-related expenses (e.g. durbars, radio announcements, health talk shows etc.). Communication equipment's (e.g. phones, computers, PA systems, projectors etc.)
Leadership, Management & Governance	Benefits/allowances for leadership training, travel for leadership and management related activities, Other cost related to leadership and management activities (e.g., facilitation, consultancy etc.).

### Data collection

Data were collected from all seven districts (three intervention and four non-intervention districts) involved in the GEHIP project from the provider perspective. Data collection was systematically done on quarterly basis from 2012–2014 by trained personnel. Cost data were entered, cleaned and analyzed using MS-EXCEL spreadsheets. In designing these templates, discussions were first held with district directors of health services, their accountants and public health nurses. The instruments were then revised taking their inputs into consideration during a one-day training session organized to foster exchanges between the research team and the relevant health managers.

### Data analysis

Cost was estimated from the perspective of the health system (provider perspective). Cost inputs were categorized as either GEHIP implementation cost or routine primary health care costs. Cost inputs were also categorized into capital or recurrent cost inputs. Inputs were considered recurrent if they had the potential of being used up within one year. These kinds of cost inputs refer to items which are mostly purchased on regular basis e.g. drugs, detergents, fuel, stationery, salaries etc. On the other hand, capital cost refers to items that last longer than a year. Examples include; vehicles, equipment, furniture, computers, and construction or renovation of CHPS buildings. The cost of these items does not generally vary with output. To gauge these investments, we first computed costs without annualizing capital costs to represent the financial cost of implementing GEHIP. Then we estimated the breakdown of these expenditures to annualize capital costs by spreading the value of capital items over their expected useful life to represent the economic cost. While the financial cost is required for budgeting purposes, the economic costs are important for program replication, planning, and implementation.

There are several alternative approaches to valuing and measuring capital costs in economic evaluations, the most widely applied approach, however, is to annualize capital outlay over the useful life of the asset to derive an estimate of its equivalent annual cost [43]. To conform with procedures conventionally used in costing studies of this nature in our context, a discount rate of 3% was employed [38, 41, 44]. This makes it possible to arrive at the value of the item consumed (depreciated) during the period under consideration. This value was then included in the cost estimate for the given period to represent the economic cost.

Finally, we apportioned costs to cost centers, such as health human resources, health service delivery, medicines & vaccines, health information, and leadership/governance building blocks.

### Ethical considerations

Ethical approval was obtained from the Ethical Review Committee of the Ghana Health Service and Institutional Review Board (IRB) of the Navrongo Health Research Centre prior to the conduct of this study.

## Results

### Background characteristics of study districts

Fig 1 provides a map of the Upper East region showing the intervention and non-intervention districts of GEHIP. Before the inception of the project in 2011, the total population in the three intervention districts was about 311,230 and this rose to 325,151 by the year 2015. The population of the four non-intervention district was about 565,096 in the year 2011 rising to about 590,373 by the year 2015. Functional CHPS zones increased from a nominal figure of 50 to 104 in intervention districts while an increase from 47 to 68 of CHPS functional zones occurred in the non-intervention districts. By 2015, two of the intervention districts and all four non-intervention districts had district hospitals. Intervention districts had 21 sub-districts while non-intervention districts put together had 42 sub-districts. However, intervention districts had more health centers (27) as against non-intervention districts with just 19 health centers. On the other hand, non-intervention districtshad more clinics (31) compared with intervention districts (7). Table 2 presents more information on health infrastructure of study districts before and after the implementation of GEHIP.

**Table 2:** Background characteristics of study districts.

**Selected background characteristics**	**Intervention Districts**						**Non-intervention Districts**							
	Bongo		Builsa District		Garu-Tempane		Talensi-Nabdam		Bolgatanga		Bawku West		Bawku East	
	2011	2015	2011	2015	2011	2015	2011	2015	2011	2015	2011	2015	2011	2015
**Population**	85,560	89,387	94,107	98,316	131,563	137,448	116,400	121,607	133,129	139,083	95,162	99,419	220,405	230,263
**Hospitals**	1	1	1	1	0	0	0	1	1	1	1	1	3	3
**Sub-Districts**	6	6	6	6	6	9	6	13	9	9	6	8	6	12
**Health Centers**	5	8	6	6	8	13	3	2	6	6	2	4	8	7
**Clinics**	1	2	1	2	4	3	4	6	6	8	6	11	12	6
**Demarcated CHPS zones**	36	36	30	15	29	30	12	29	18	23	17	21	26	17
**Functional CHPS zones**	14	36	10	30	26	38	12	24	10	16	13	18	12	10
**Total Health Staff**	150	289	157	256	101	142	103	308	238	398	167	274	284	420

NB: In 2013, the Government of Ghana created new districts by splitting Builsa District into two, Talensi-Nabdam into two and Bawku East into three districts. Results in this paper, however, reports on the original area for districts before there were partitioned.

### Financial cost of implementing GEHIP

The financial cost of implementing GEHIP from 2012 to 2014 is shown in Table 3. Financial cost aims to show the actual money spent on implementation, and to that end, capital cost is not annualized. The overall financial cost of implementing GEHIP in the three districts during the period under study was $569,156 representing $1.79 per capita. Separation of these costs by year yields an estimated financial cost of $0.63, $0.78 and $0.38 per capita in 2012, 2013, and 2014, respectively (see Table 3).

**Table 3:** Financial cost of implementing GEHIP (2012–2014).

Year	Financial cost ($)	Population	Financial cost per capita ($)
2012	199,587	314,964	0.63
2013	248,588	318,744	0.78
2014	120,981	321,931	0.38
Total	569,156		1.79

Table 4 presents the financial cost of implementing GEHIP by health system building blocks. Overall, improving health service delivery contributes as high as 62.5% of the total financial cost, human resources cost contributing 16.9%, medications cost contributing 8.3%, leadership and its related activities contributed 7.6% of the total financial cost whiles 4.7% of total cost was related to the cost of reforming health information systems.

**Table 4:** Financial cost of GEHIP by health system building blocks.

Cost categories (cost centers)	2012		2013		2014		Total	
	Cost ($)	%	Cost ($)	%	Cost ($)	%	Cost ($)	%
**Human Resources**	37,563	18.8	41,682	16.8	17,158	14.2	96,403	16.9
**Health Service Delivery**	104,412	52.3	156,952	63.1	94,126	77.8	355,490	62.5
**Medicines**	25,147	12.6	22,345	9.0	-	-	47,492	8.3
**Health Information**	11,164	5.6	11,227	4.5	4,187	3.5	26,577	4.7
**Leadership/Governance**	21,301	10.7	16,382	6.6	5,510	4.6	43,193	7.6
**Total**	199,587	100.0	248,588	100.0	120,981	100.0	569,156	100.0

### Economic cost of implementing GEHIP and routine primary healthcare

Table 5 presents the results of the economic cost of implementing GEHIP and routine primary health care in intervention and non-intervention districts. The total primary healthcare economic cost in the intervention districts was $3,714,077, $4,542,589 and $4,578,809 respectively for the years 2012, 2013 and 2014. The total primary healthcare economic cost in the non-intervention districts was $7,014,984, $8,375,598 and $6,805,317 for 2012, 2013 and 2014 respectively. GEHIP’s economic cost as a percentage of total primary health care cost was 3.6% in 2012, 3.2% in 2013 and 1.6% in 2014.

**Table 5:** Economic cost by intervention and non-intervention districts (2012–2014).

Year		Intervention Districts			Non-Intervention Districts		
		Cost	(%)	Per capita	Cost	(%)	Per capita
**2012**	GEHIP implementation cost	131,757	3.6	0.4	-	-	
	Routine cost	3,582,320	96.4	11.4	7,014,984	100	12.3
	Total cost	3,714,077	100	11.8	7,014,984	100	12.3
**2013**	GEHIP Implementation cost	144,908	3.2	0.5	-		
	Routine cost	4,397,681	96.8	13.8	8,375,598	100	14.5
	Total cost	4,542,589	100	14.3	8,375,598	100	14.5
**2014**	GEHIP Implementation cost	74,124	1.6	0.2	-		
	Routine cost	4,504,685	98.4	14.0	6,805,317	100	11.6
	Total cost	4,578,809	100	14.2	6,805,317	100	11.6

Overall, the per capita economic cost of primary healthcare delivery was $11.8, $14.3 and 14.2 in intervention districts for 2012, 2013 and 2014 respectively while that of non-intervention districts was $12.3, 14.5 and 11.6 for the same period. The total economic cost of implementing GEHIP over the three-year period was $ 337,393. This translates into a per capita cost of $1.46 in Bongo district, $1.02 in Builsa district and $ 0.86 in the Garu-Tempane district, and the average per capita cost is $1.07. The economic cost of implementing GEHIP is presented according to WHO systems building blocks in Table 6. Health service delivery accounted for the highest cost component of 37.5%, human resources accounted for 28.6% while medicines was 13.6% of economic cost. Health information and leadership related activities accounted for 7.5% and 12.8% respectively.

**Table 6:** Economic cost of implementing GEHIP program (2012–2014).

Cost categories (cost center)	Bongo District		Builsa District		Garu-Tempane District		Total	
	Cost ($)	%	Cost ($)	%	Cost ($)	%	Cost ($)	**%**
**Human Resources**	51,221	40.6	16,775	17.3	28,407	24.9	96,403	28.6
**Health Service Delivery**	32,738	25.9	53,061	54.7	40,845	35.8	126,643	37.5
**Medicines**	12,383	9.8	18,489	19.0	15,038	13.2	45,911	13.6
**Health Information**	9,475	7.5	3,723	3.8	12,045	10.6	25,243	7.5
**Leadership/Governance**	20,402	16.2	5,041	5.2	17,751	15.6	43,193	12.8
**Total**	126,219	100	97,089	100	114,085	100	337,393	100

Over the three years, the cost of implementing GEHIP as a percentage of total primary health care cost in intervention districts was 3.1% in Bongo district, 2.8% in Builsa district and 3.5% in Garu-Tempane district.

Considering the health system building blocks, service delivery and medicines cost more in the non-intervention than in the intervention districts combined (both routine and implementation cost). However, the cost of human resources, health information, and leadership/governance were greater in the intervention districts than that of the non-intervention districts. This is probably due to the fact that GEHIP program enabled intervention districts to strategically plan and prioritize their programs and expenditure to include all building blocks; a situation that was lacking in the non-intervention districts.

**Fig 2:**
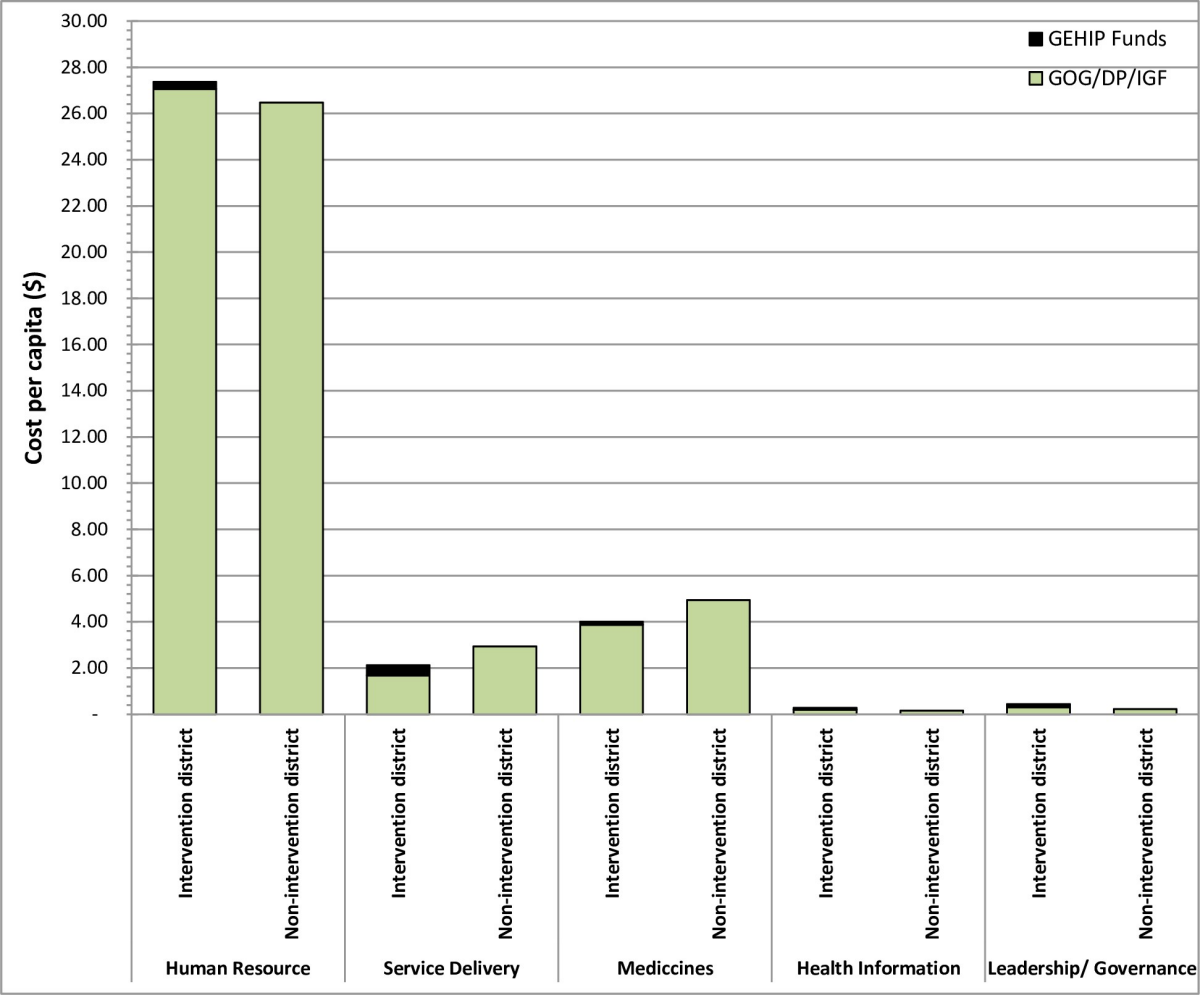
Cost per capita by intervention & non-intervention districts.

Fig 2 shows the total cost per capita by health system building blocks. The total per capita incremental cost on human resource was $0.32, health service delivery was $0.44 while medicines and medical supplies was $0.14, health information building block was $0.08 and leadership/governance wa $ 0.14 per capita.

## Discussion

This paper has analyzed the cost of implementing the GEHIP program in the Upper East region of northern Ghana according to categories of resources that have been expended on health system components. It was found that the per capita cost of implementing GEHIP over a three-year period was $1.79 and $1.07 for financial and economic cost respectively. This was a modest investment given the fact that GEHIP accelerated CHPS coverage from 20 percent in the baseline to 100 percent CHPS coverage in targeted districts, a pace of implementation that was double the rates observed in comparison districts [17]. In addition, there was a nearly 50% greater reduction in infant mortality in intervention districts, compared to non-intervention districts [28]. Therefore, GEHIP had a major impact on early infant survival. Its contribution as a percentage of total primary healthcare cost in implementation districts was however low, ranging from 2.8% in Builsa district to 3.5% in Garu-Tempane district. The total financial cost of implementing GEHIP was $569,156 while its equivalent economic cost was $337,393. As a percentage of total health investment, however, the economic cost of implementing GEHIP was a marginal investment: 3.6% of total primary healthcare cost in 2012, 3.2% in 2013 and 1.6% in 2014. It was also found that the overall per capita cost of service delivery and medicines was slightly higher in non-intervention districts compared to intervention districts even when implementation cost was combined with routine cost. This is most likely due to differences in health facilities and staff of districts that existed even before GEHIP was implemented. For example, interventions districts were found to be relatively more remote and isolated compared with the non-intervention districts. Therefore, non-intervention districts had more highly trained health personnel that favored more curative than preventive care hence the higher per capita cost in service delivery and medications.

The cost differentials in the relative weight of the WHO health system blocks observed across intervention districts is as a result of the different prioritization of needs. GEHIP provided leadership trainings and guidance to district health managers at the inception of the project. This allowed each district team to plan and draw annual budgets based on their needs. Recognizing the different levels of CHPS implementation and health infrastructure situation of districts at the onset of GEHIP (see Table 2), the program allowed for flexibility in the allocation of funds within a common framework of GEHIP’s set of interventions. The implication of these differences in cost suggest that health system strengthening initiatives are likely to be more effective if flexibility in funds allocation is allowed for addressing contextual priorities identified by system managers. It was also observed that the proportionate cost by WHO building blocks differed between financial costs and economic cost. For instance, health service delivery and human resources accounted for 62.5% and 16.5% of the financial cost of implementing GEHIP respectively. However, when economic cost was estimated by spreading capital cost items over their useful life years, the proportion of cost on health service delivery reduced to 37.5% while that of human resources increased to 28.6%. This has different implications for program budgeting, planning, implementation, and replication. For budgeting purposes, consideration should be given to financial cost estimates. However for program planning purposes the economic cost estimates should be given prominence.

Although there is evidence that community-based strategies aimed at stimulating both demand and supply sides of health care services can be cost-effective [45], there is limited evidence on how the additional cost of such initiatives will be borne by the health system at large. Our results show that the marginal per capita cost of implementing GEHIP is relatively low. The cost of GEHIP program combined with routine cost in intervention districts was still lower compared with the cost of delivering primary health services in the non-intervention districts. GEHIP’s contribution as a percentage of total health system cost in intervention districts was estimated to be just about 3% of the entire cost of primary health care delivery.

These costs are also low relative to the cost of services provided by the Navrongo Experiment over the 1996–2003 periods. Unadjusted for inflation, the initial start-up cost of the Navrongo experiment was US$ 1.92 per capita [14]. Comparing this investment with the $1.07 per capita associated with GEHIP suggests that the cost of improving community-based primary health care program that is already underway may be lower than the cost of initiating a similar program from scratch.

However, it is noteworthy that the cost of implementing GEHIP was low because the implementation of functional Community-based Health Planning and Services (CHPS) under GEHIP was linked to community engagement and volunteer construction by communities, rather than relying on expensive construction costs. This approach meant that services could begin without waiting for expensive construction. And, where the strategy worked best, District Health Management Teams (DHMTs) targeted their construction funds on communities where volunteer efforts permitted implementation to occur. In this manner, expensive investment in construction became an incentive for implementation rather than a barrier to implementation. Thus, implementing CHPS is inexpensive if community engagement is properly pursued. For this reason, GEHIP could achieve total coverage of CHPS in intervention districts compared to non-intervention districts where only half as much coverage was achieved [28]. However, to sustain such gains over time, health managers must continue to foster community participation through the initiation of active community health management teams and community volunteer groups that ensure continuous community involvement in the planning and delivery of primary healthcare services.

The pronounced impact of such small marginal investments invites careful deliberations on the implications of GEHIP for decision-making processes of health managers, policy makers and development partners in the health sector of Ghana. First, such modest investments suggest that these costs are replicable because the additional cost of fostering community engagement, district and sub-district leadership, and inter-sectorial partnership are inexpensive. But most importantly, GEHIP attests to the economic value of social engagement as a means of accelerating CHPS coverage and maximizing its impact on primary health care service delivery and health service benefits. Investment in personnel, equipment, modalities, and the WHO health system building blocks as program components are critical to systems strengthening and community health systems development. But open systems investment in community engagement, exchanges between community leaders, and consensus building are catalytic inputs that GEHIP mastered with dramatic results at minimal costs. Program component investment is essential, but GEHIP marginal investment in leadership capacity building has improved CHPS scale-up.

This study employed basic principles for costing health care services as recommended by the WHO [38] and used by similar studies that have been conducted in other settings [39–42]. Our adoption of WHO system building blocks as analytical cost components of the system is, however, novel and could be applied by future studies for health system costing. We would aim to expand this analysis in our future endeavors with the possibility of performing a cost-effectiveness analysis of this intervention in order to provide more evidence for guiding resources allocation decisions for community-based primary healthcare initiatives.

### Study limitations

Our cost estimates were based on expenditures in the implementation period of GEHIP which might not reflect the full cost of producing outputs. For example, the value added by the utilization of existing buildings could not be factored into the cost estimates. Also, as previous studies on this subject are limited, it was difficult to meaningfully compare findings of our study to previous studies. Despite these limitations, the methods applied in this study are reliable and can be adapted for future costing of community-based primary health care services initiatives.

## Conclusion

This study contributes to an understanding of the additional cost of improving leadership capability for developing social engagement aimed at implementing community-based primary health care programs in Ghana. We found the additional cost of implementing GEHIP to be relatively low. The low cost of this initiative attests to the importance of directing resources to flexible mechanisms that enable local managers to make decisions that build local ownership and participation in the implementation of community-based primary health care. Given the success of GEHIP in improving geographical access to healthcare and inducing both supply and demand for health care and ultimately impacting on under-five mortality, GEHIP has had a catalytic effect on the efficient use of routine resources, an outcome of the program that policy review, replication, and scale-up should be mindful of.

## Supporting information

S1 File Data collection template. (XLS)

S2 File Data set. (XLSX)

## Abbreviations

GEHIP: Ghana Essential Health Intervention Program
CHPS: Community-based Health Planning and Services
CHFP: Community Health and Family Planning
CHOs: Community Health Officers
MDG: Millennium Development Goals
WHO: World Health Organization
GHS: Ghana Health Service
NHRC: Navrongo Health Research Center
GOG: Government of Ghana
DPF: Donor Pool Funds
IGF: Internal Generated Funds
PHC: Primary Health Care
UER: Upper East Region
DHMT: District Health Management Team
RHD: Regional Health Directorate
